# The Risk of Drug Abuse among Preschool Students in Phuket, Thailand

**Published:** 2019-03

**Authors:** Kusuma SWANGPUN, Manop KANATO, Poonrut LEYATIKUL

**Affiliations:** 1. Community Health Development Program, Faculty of Medicine, Khon Kaen University, Khon Kaen, Thailand; 2. Department of Community Medicine, Faculty of Medicine, Khon Kaen University, Khon Kaen, Thailand; 3. Department of Occupational Health, Faculty of Public Health, Vongchavalitkul University, Nakhon Ratchasima, Thailand

**Keywords:** Drug abuse risk, Prevalence, Preschool students

## Abstract

**Background:**

There have been scientific studies on risk and protective factors associated with child outcomes. However, how risk factors interact to produce outcomes is not clearly understood. We assessed the association between school location, grade, and drug abuse risks among preschoolers.

**Methods:**

This cross-sectional study included 3353 participants aged 2–6 yr (mean: 4.6, SD: 1.03) enrolled in childcare centers and kindergarten classes in 2016 in Phuket, Thailand. The risk of drug abuse was measured using questionnaires, completed by teachers and caregivers. Data were analyzed using descriptive statistics, odds ratio with 95% confidence intervals and Chi-square.

**Results:**

The prevalence of drug abuse risk factors was as follows: sleep problems=1.28%, aggression=1.10%, attention-deficit hyperactivity disorder (ADHD)=3.61%, learning disabilities (LDs)=4%, family members’ substance abuse=12.53%, parents’ changing marital status=6.53%, conflict between parents/guardians=1.88%, family poverty=3.37%, and easy access to drugs in the community=5.16%. Sleep problems in children were significantly related to family members’ substance abuse, family poverty, and easy access to drugs in the community (PS<.05). LDs were significantly related to parents’ changing marital status, conflict between parents/guardians, family poverty, and easy access to drugs in the community (PS<.05). ADHD was significantly related to family members’ substance abuse, parents’ changing marital status, conflict between parents/guardians, and family poverty (PS<.05).

**Conclusion:**

The prevalence of drug abuse risks among preschoolers was 25.86%. Multidisciplinary teams should provide appropriate interventions.

## Introduction

Drug abuse is a worldwide problem. The United Nations Office on Drugs and Crime estimated that 5% of the global population aged 15–64 yr used drugs at least once in 2015 (1). The National Survey of Drug Use and Health, which examined the age of onset among individuals across the United States (U.S.), revealed that drug use commences at the age of 12 yr (inappropriate prescription drugs use, alcohol, and tobacco) (2).

In Thailand, drug abuse has been reported as a social problem at least since 1360 (3). The Office of the Narcotics Control Board has reported on drug use and the complexity thereof, particularly among youths (4). The latest national household survey on substance use indicated that approximately 2.96 million people or 58.16 per 1000 population used drugs at least once in their lifetime (5). Furthermore, 1.4 million people had used one or more substances within the past year. Amphetamine-type stimulants and plant-based products were the most popular substances (5). New psychoactive substances (i.e., ice) were introduced more recently (6).

A study showed most drug users to be young people aged 15–24 yr, with methamphetamine as the predominantly used substance group (7). Additionally, drug use in younger children is a concern. Numerous drug-abusing young children (aged <12 yr) received medical treatment between 2006 and 2015 (8).

A substantial number of scientific studies on risk and protective factors associated with child outcomes have been conducted since the mid-1980s (9). Preschool risk factors associated with drug abuse problems have also been examined. For example, sleep problems have been shown to be related to early-age alcohol consumption in both sexes and, among boys, to be related to smoking and to predict marijuana use (10). Chronic stress among young children is also associated with alcohol consumption during adolescence and drug abuse in adulthood (11). Furthermore, anxiety in children has been shown to predict the age of substance abuse in boys (12). In addition, children’s depression positively correlates with alcohol use. Children with moderate to severe depression vastly outnumber those with mild depression (13).

Antisocial behaviors such as aggression and delinquency in preschool (among children aged 3–5 yr) are also associated with alcohol consumption from a young age (14) and marijuana use, especially among young boys (15). Furthermore, children with attention-deficit hyperactivity disorder (ADHD) are more likely to use nicotine and other addictive substances (16). In addition, learning disabilities (LDs) can also affect children’s development and are associated with inappropriate behavior (17–19).

Risk factors at family, society, and environmental levels may also lead to future drug abuse. In a longitudinal study with children aged 4–14 yr, key risk factors associated with children’s future health problems included abuse (i.e., physical, psychological, and sexual), abandonment, caregivers’ substance abuse, caregivers’ depression, and a family member having a criminal record (20,21). School/community factors such as peers’ involvement in risky/problem behaviors and access to drugs in schools and the community are also associated with adolescents’ drug use (22–24). Some signs of risk can be seen as early as six years of life or lower. Therefore, data of pre-schooler is necessary.

The objective was twofold: to explore the prevalence of drug abuse risks among preschoolers and to assess the association between school location, grade, and drug abuse risk.

## Materials and Methods

### Design

The present study employed a cross-sectional design in all 28 childcare centers and kindergartens (organized by the local government) in Muang Phuket, Thailand.

### Population

Participant recruitment took place during semester 1 of the 2016 academic year. Of all 3654 students aged 2–6 yr, 3,353 participated (91.76%).

### Tools

A summated rating scale on risk assessment for drug abuse problems among preschoolers was developed by teachers/caregivers and experts/clinicians. Items with item-objective congruence (IOC)=0.67 or above were selected. Finally, 85 items on personal factors, family-social, and community factors were selected. The personal factors include 8 subgroups, and each subgroup has 10 questions for measuring sleep problems, stress, aggression, anxiety, depression, attention-deficit hyperactivity disorder, learning disabilities, abuse, and neglect. The cut-off score at risk of each personal factor is 6 scores or more. The family-social and community factors include 5 questions for measuring family members’ substance abuse, parents’ changing marital status, conflict between parents/guardians, family poverty, and easy access to drugs in the community. Additionally, the cut-off score at risk of each family-social and community factor is 1. Five content experts were asked to assess content validity index (CVI), which was 0.93. Two observers were asked to assess 150 preschoolers in Songkhla. Kappa was 0.91 for inter-observer’s reliability.

### Data collection

All teachers/caregivers and parents who had been close to their children for at least 2 months provided informed, voluntary consent to participate in the study and completed the forms from Apr to May 2016.

### Data analyses

Double data entry was conducted, and the data validated. Data exploration was performed to correct for out-of-range values, outliers, and missing values. The data set was analyzed using descriptive statistics, a chi-square test, and odds ratio with 95% CI.

### Ethical approval

The present study was approved by the Human Research Ethical Committee of Khon Kaen University. Written, informed consent was obtained from teachers/caregivers and from children’s parents/guardians.

## Results

Overall, 3353 students were assessed for drug abuse risks (Table 1). The prevalence of each drug abuse risk factor among the preschoolers is presentedThe highest prevalence (5% and above) were family members’ substance abuse, Parents’ changing marital status, and Easy access to drugs in the community (Table 2). Around a quarter of the sample present with one or more risk factors (Table 3). As in Table 4, grade is statistically significant (*P*<0.05) related to risk.

**Table 1:** Participants’ demographic characteristics (N=3353)

***Characteristics***	***n***	***%***
Grade		
Pre-kindergarten	1,470	43.84
Kindergarten	1,883	56.16
School location		
In Phuket municipality	1,512	45.09
Outside Phuket municipality	1,841	54.91
Age		
2 yr	7	0.21
3 yr	453	13.51
4 yr	1,350	40.26
5 yr	633	18.88
6 yr	910	27.14

**Table 2:** Prevalence of drug abuse risks among preschoolers (N=3353)

***Variable***	***Drug Abuse Risk (%)***	***Mean***	***Standard Deviation***	***Range***
Sleep problems	43 (1.28)	1.00	1.49	0–9
Stress	26 (0.78)	0.60	1.19	0–8
Aggression	37 (1.10)	0.40	1.07	0–10
Anxiety	23 (0.69)	0.60	1.21	0–9
Depression	30 (0.89)	0.39	1.03	0–10
Attention-deficit hyperactivity disorder	121 (3.61)	0.99	1.76	0–10
Learning disabilities	134 (4.00)	0.99	1.85	0–10
Abuse and neglect	1 (0.03)	0.06	0.33	0–6
Family members’ substance abuse	420 (12.53)	0.13	0.33	0–1
Parents’ changing marital status	219 (6.53)	0.07	0.24	0–1
Conflict between parents/guardians	63 (1.88)	0.02	0.13	0–1
Family poverty	113 (3.37)	0.03	0.18	0–1
Easy access to drugs in the community	173 (5.16)	0.05	0.22	0–1

**Table 3:** Percentage of preschoolers with drug abuse risk factors (N=3353)

***Students’ drug abuse risk factors***	***n***	***%***
No. of risk factors	2,686	75.60
1 risk factor presented	538	16.05
2 risk factors presented	201	5.99
3 risk factors presented	74	2.21
4 risk factors presented	35	1.04
5 risk factors presented	14	0.42
6 risk factors presented	4	0.12
7 risk factors presented	1	0.33
Total of risk persons	867	25.86

**Table 4:** Association between school location, grade, and drug abuse risks (N=3353)

***Variable***	***Risk (%)***	***Normal (%)***	***χ^2^***	***P-value***	***OR***	***95% CI***
School location						
In Phuket municipality	395 (45.6)	1,117 (44.9)	0.079	0.78	1.026	0.878–1.198
Outside Phuket municipality						
Grade level	472 (54.4)	1,369 (55.1)				
Pre-kindergarten	464 (53.5)	1,006 (40.5)	43.94	< 0.01	1.694	1.450–1.979
Kindergarten	403 (46.5)	1,480 (59.5)	4			

We examined the association of three key risk factors (i.e., sleep problems, LDs, and ADHD) with factors in the community and the family social environment. Sleep problems were significantly related to family members’ substance abuse, family poverty, and ease of access to drugs in the community. LDs were significantly related to parents’ marital status, conflict between parents/guardians, family poverty, and ease of access to drugs in the community. ADHD was significantly related to family members’ substance abuse, parents’ marital status, conflict between parents/guardians, and family poverty (Table 5).

**Table 5:** Association between environmental factors and sleep problems, LDs, ADHD (N=3353)

***Environmental Factors***	***Behavior problems***		***Yates’ chi-square***	***P-value***
	***Risk n (%)***	***Normal n (%)***		
Family members’ substance abuse	Sleep problems			
No	2,904 (87.73)	29 (67.44)	14.15*	< .001
Yes	406 (12.27)	14 (32.56)		
Parents’ changing marital status				
No	3,097 (93.56)	37 (86.05)	2.80	.09
Yes	213 (6.44)	6 (13.95)		
Conflict between parents/guardians				
No	3,249 (98.16)	41 (95.35)	0.61	.43
Yes	61 (1.84)	2 (4.65)		
Family poverty				
No	3,203 (96.77)	37 (86.05)	11.87*	< .001
Yes	107 (3.23)	6 (13.95)		
Easy access to drugs in the community				
No	3,143 (94.95)	37 (86.05)	5.18*	.02
Yes	167 (5.05)	6 (13.95)		
Family members’ substance abuse	ADHD			
No	2,840 (87.87)	93 (76.86)	11.92*	< .001
Yes	392 (12.13)	28 (23.14)		
Parents’ changing marital status				
No	3,031 (93.78)	103 (85.12)	12.94*	< .001
Yes	201 (6.22)	18 (14.88)		
Conflict between parents/guardians				
No	3,179 (98.36)	111 (91.74)	24.29*	< .001
Yes	53 (1.64)	10 (8.26)		
Family poverty				
No	3,131 (96.87)	109 (90.08)	14.50*	< .001
Yes	101 (3.13)	12 (9.92)		
Easy access to drugs in the community				
No	3,070 (94.99)	110 (90.91)	3.06	.08
Yes	162 (5.01)	11 (9.09)		
Family members’ substance abuse	LDs			
No	2,394 (81.87)	77 (77.00)	1.23	.268
Yes	530 (18.13)	23 (23.00)		
Changing marital status of parents				
No	2,689 (91.96)	82 (82.00)	11.25*	< .001
Yes	235 (8.04)	18 (18.00)		
Conflict between parents/guardians				
No	2,852 (97.54)	91 (91.00)	13.45*	< .001
Yes	72 (2.46)	9 (9.00)		
Family poverty				
No	2,752 (94.12)	80 (80.00)	30.08*	< .001
Yes	172 (5.88)	20 (20.00)		
Easy access to drugs in the community				
No	2,813 (96.20)	85 (85.00)	27.66*	< .001
Yes	111 (3.80)	15 (15.00)		

## Discussion

The present study aimed at determining the influence of behavioral and environmental factors on the risk of drug abuse among preschoolers in Thailand. The prevalence of drug abuse risks in preschoolers was 25.86%, which was greater than that reported (i.e., 11.9%) in Bangkok (25) and another in Thailand on children aged 5–16 yr (i.e., 20.15%) (26). However, the current study’s prevalence was lower than that found in a study on children aged 8–11 yr in Bangkok (i.e., 37.58%) (27).

Specifically, the prevalence of LDs was the highest, followed by that of ADHD and sleep problems. The following behavioral problems were reported less often than in a study among children aged 1–5 yr (north of Bangkok): sleep problems=24.7%, aggression=21.7%, and emotional problems=27.7% (28). Furthermore, an earlier study reported the prevalence of ADHD among preschoolers aged 4–6 yr to be 24.0% (25).

The prevalence of behavioral and emotional problems in Thai children differs from that reported in international studies, as shown by a study of psychiatric disorders in children in Canada, where the rate of psychiatric disorders was 18.1% and ADHD was 8.3% (29). However, LDs, ADHD, and sleep problems are common in children and may affect long-term learning ability (28). Parents should support children’s cognitive development (e.g., executive functioning) to promote positive thinking and behavior, which may reduce the children’s risk of conduct disorders and drug abuse (30–32).

Consistent with earlier international research, we found that family poverty was related to behavioral and emotional problems among preschoolers (33).

### Limitation

The present study involved subjects living in a specific area of Muang Phuket, therefore it may not represent characteristic of varied populations. Teachers/caregivers and parents may assess students with LDs and ADHD differently because parents cannot observe students’ behaviors during school hours. However, teachers/caregivers and parents who answer the questionnaire should stay close to their children for at least two months during each semester.

## Conclusion

We revealed that a considerable number of pre-schoolers may be at risk for future drug abuse. LDs were the most prevalent behavioral risk factor; family members’ substance abuse was the most prevalent environmental risk factor; and family poverty was related to sleep problems, ADHD, and LDs. In addition, pre-kindergarten children had a higher risk of drug abuse than did kindergarten students.

Proactively monitoring preschoolers’ drug abuse risks may facilitate early detection and prevent drug abuse problems at the primary school level. Multidisciplinary teams should provide appropriate interventions to at-risk students at the individual, family, school, and community levels.

## Ethical considerations

Ethical issues (Including plagiarism, informed consent, misconduct, data fabrication and/or falsification, double publication and/or submission, redundancy, etc.) have been completely observed by the authors.
